# Probiotics in allergic rhinitis

**DOI:** 10.1590/S1808-86942011000100022

**Published:** 2015-10-19

**Authors:** Janaina Cândida Rodrigues Nogueira, Maria da Conceição Rodrigues Gonçalves

**Affiliations:** 1 MD; MSc; 2 Professor. Coordinator of the Graduate Program in Nutriton - UFPB

**Keywords:** bifidobacterium, lactobacillus, rhinitis, probiotics

## Abstract

Probiotics are live microorganisms used as supplementary food, usually lactic acid bacteria that can change either the composition and/or the metabolic activities of the gut microbiota modulating the immune system in a way that benefits the person's health.

**Aim:** to review the use of Probiotics (Lactobacillus and Bifidobacterium) in allergic rhinitis patients.

**Materials and Methods:** Pubmed original articles were used as data source.

**Results:** results indicate that probiotics, Lactobacillus and Bifidobacterium appear to prevent allergy recurrences, alleviate the severity of symptoms and improve the quality of life of patients with allergic rhinitis. This happens because of the immune system modulation through the induction of cytokine production which cause a dominant TH1 response in allergic patients by modulating the TH1/TH2 balance effect.

**Conclusion:** The use of probiotic bacteria could be an effective and safe way to prevent and/or treat allergic rhinitis, but its underlying mechanisms remain unclear. Therefore, clinical studies using probiotics and dietary intervention should be the focus of future research to enable a more widespread use.

## INTRODUCTION

Allergic rhinitis is a nasal inflammatory disease which manifests when a sensitized individual comes in contact with foreign substances, called antigens. It is very frequent in today's world, affecting individuals of both genders and all ages. It may manifest from mild symptoms to symptoms which reduce the individual's quality of life and productivity, either in a temporary or permanent basis when there is not proper treatment^1^.

Today, both in developed countries as well as in those developing ones, we notice a different disease profile when compared to that of previous decades, with a predominance of allergic, autoimmune and chronic inflammatory diseases instead of their infectious counterparts. Part of this phenomenon is due to the “Hygiene Hypothesis”, according to which changes in the life styles of people, such as in food, consumption of industrialized products rich in preservatives and other chemical products, as well as the reduction of contact children would have with microorganisms because of better hygiene and vaccination, brought about changes to the individuals' immune characteristics^2^.

When a foreign substance comes in contact with the body, it is phagocyted and processed by the antigen-presenting cells. These cells expose this antigen on its membrane, with its histocompatibility complex (HLA) - class II, which, when it interacts with the receptor on the T CD4+ cell membrane promotes the proliferation of a specific clone of type 1 or type 2 T helper cells, which according to the secreted cytokines will enable a type of immune reaction^3^.

The T helper 1 cells produce TNF Beta (Tumor Necrosis Factor) and Interferon C which activates the cells involved in cell immunity, in other words, on the body's defense against intracellular organisms such as viruses and other microorganisms which infect the inside of the cell, defective and cancer cells. The T helper 2 cell line produce IL-4, IL-5, IL10, IL13 which stimulate cells to participate in the allergic reaction, such as IgE secreting B cells, mast cells and eosinophils^4^.

According to some authors, what would explain the “Hygiene Hypothesis” would be a higher predominance of the Th2 cell line in comparison to the Th1, where the change in intestinal microbiota can be one of the factors involved^5^.

Studies have shown that the bacteria present in the intestinal micro flora play a role in the Th1/Th2 balance, and its modulation can promote the control of infectious and immune processes. This modulation can be carried out through the use of probiotics, which are supplements of microorganisms. The probiotics concept has been around for more than 100 years. Between the XIX and XX centuries, Moro isolated *Lactobacillus* in the feces from infants fed maternal milk, and Tissier found the *bifidobacteria*; in the early XX century, Metchnikoff reported the high longevity of Bulgarian peasants who drank fermented milk, and such product is today recommended by the World Health Organization^2^.

The main probiotics are *Lactobacillus acidophilus, Lactobacillus casei, Lactobacillus plantarum, Lactobacillus reuteri, Lactobacillus rhamnosus, Lactobacillus paracasei, Bifidobacterium bifidum, Bifidobacterium breve, Bifidobacterium infantis, Bifidobacterium lactis, Bifidobacterium longum, Bifidobacterium adolescentis, Saccharomyces bourlardii, Propionibacterium freudenreichii*; and also *Escherichia, Enterococcus* and *Bacillus* and the *Saccaromyces boulardii* fungus. Notwithstanding, despite being beneficial to the body and being frequently added to children food, the *Lactobacillus bulgaricus* and *Streptococcus thermophilus* are not considered probiotics^2^.

In order for a probiotic agent to be satisfactory, the component bacteria must be natural dwellers of the GI tract, must be resistant to gastric secretions and bile, have a precise taxonomic identification, colonize the intestinal mucosa, be active in the food before its consumption and be continuously ingested. Its minimum dose is 106 UFC6.

Studies have assessed the benefits of using probiotics to manage allergies in improving the function of the native intestinal microbiota^6^. Based on these facts, this bibliographic review aimed at assessing the importance of using probiotics in allergic rhinitis through reading the scientific literature indexed in the Pubmed between the years of 2000 and 2008.

## MATERIALS AND METHODS

This study is a bibliographic review on the topic of probiotic and allergic rhinitis. The source of the information used was the Pubmed database, between 2000 and 2008. We included preferably clinical trials, meta-analyses and review papers in Portuguese, English and Spanish, as well as papers which topic discussed was the clinical or experimental use of *Lactobacillus* and *Bifidobacterium* on allergic rhinitis, and we took off those studies concerning other sources of allergy. The keywords used in the respective languages were: “probiotics, allergic rhinitis, *Lactobacillus*, *Bifidobacterium*”.

When the keyword *Bifidobacterium* was studied, 1963 papers were found, increasing the number to 7012, when the keyword used was *Lactobacillus.* These findings suggest a great interest in probiotics, especially concerning *Lactobacillus*, very likely because of its broad use as food supplement and because of the growing interest in its use for health promotion purposes. When we cross-referenced rhinitis and *Lactobacillus* keywords, rhinitis and *Bifidobacterium* and rhinitis and probiotics, these figures decrease to 26, 10 and 46 papers, respectively.

## LITERATURE REVIEW

The intestinal barrier is our strongest body barrier against microorganisms and foreign substances. Disorders in the intestinal microbiota change the permeability and reduce the intestinal selectivity to macromolecules, and such phenomenon is known as “leaky gut,”. Probiotics can reinforce the intercellular junctions in the GI tract, making the intestine “less leaky” - as well as increasing the cytokines which stimulate lymphocytes of the Th1 line, reestablishing oral tolerance and reducing the allergic response^7,^^8^. Then, it is understood that the intestinal flora make up is important in the immune response balance.

A study assessing children from countries of low incidence of allergic diseases, compared to children from countries of low incidence of these diseases, showed that there were differences in the intestinal flora makeup of the children with a predominance of *Clostridium* and *S. aureus* in the former and *Lactobacillus*, *Bifidobacteria* and *Enterococos* in the latter^9^.

Numerous studies have reported on the antiallergic activity of *Lactobacilli*, with only a few studies involving *Bifidobacteria*, and such activity was specific for species concerning both bacteria. However, what one can notice in these studies is that both the probiotics of the *Lactobacillus* genus as the *Bifidobacterium* suppressed the allergic response, promoting modulation and regulation of the immune system^10^.

Aldinucci et al., 2002, investigated 20 volunteers: 13 with allergic rhinopathy and 7 healthy individuals - who made up the control group, concerning the effects of consuming yogurt with *Lactobacillus delbruekii*, sub bulgaricus and *Streptococcus thermophiles* for four months, in other words two types of probiotic agents: one L*actobacillus acidophilus* and one *bifidobacterium* or skim milk. Of the 13 individuals, 7 received 450g of yogurt and 6 were given 450g of partial skim milk, and the regular nutrition was maintained. We assessed the following parameters before and after: rate of mononuclear cell proliferation and its release of Interferon Gama (INF- γ) and Interleukin 4 (IL-4). In order to assess allergic rhinopathy, we used nasal function tests (anterior rhinometry, acoustic rhinometry), the Prick test, specific nasal provocation test (NPT), dosage of specific IgE in the blood, assessment of symptoms and nasal mucociliary transport test. The patients who were fed yogurt had improvements in their symptoms and in nasal mucociliary transport, and the other parameters remained unaltered; as far as cell culture goes, there was a reduction in IL-4 secretion and more TNF-γ secretion; without changes to the proliferation rate. According to the authors, these findings indicate a beneficial effect of using this type of foods stuff^10^.

The dendritic cells are considered the most efficient antigen-presenting cells, with an important participation in the onset of the primary immune response and in the development of the secondary response. These cells can be modulated by the probiotic agents and this could be possible due to specific membrane receptors called “tolllike” for the bacteria. Christensen et al., 2002, showed that different species of *Lactobacillus* cause different forms of activation of the dendritic cells^11^.

Kimoto et al., 2004, carried out a study in order to assess the immune modulatory activity of 15 strains of *Lactobacillus*. Initially, they carried out an *in vitro* assessment of the cytokine induction capacity of murine macrophages of the J774 line in culture. In this experiment, 6 strains of *Lactobacillus* induced the production of IL2, IL6, TNF-α in a species-specific mode. Of these strains, G50 was the one that most induced cytokine, thus being isolated for further studies. They also analyzed the cytokine-induction capacity of macrophages in culture, induced by strains of L. lactis subsp. Lactis G50 killed by heat to assess its activity even in the unfeasible form. It was observed that this strain continued to induce cytokine production, suggesting that such activity is associated with elements on the bacterial wall. We can also notice that the unfeasible forms stimulated a greater production of IL12 and lower of IL6, when compared to feasible strains^12^.

The same authors assessed the *In vivo* activity of the G50 strain of Lactobacillus taken orally during 7 days, by BALB/c mice and later on analyzed its spleen cells, and they observed a growth in the synthesis of IL12 and interferon Gama and mild reduction of IL4 and IL6, in relation to controls. The effect of *Lactobacillus*, in the production of *In vivo* antibodies, was also studied in the immunization of mice with OVM (ovomucoid) measuring the level of antibodies in the plasma. The specific IgE rate for the antigen used (OVM) in the serum of those who received *Lactobacillus* G50 caused a significant reduction (p<0.01) in comparison to the controls, as well as that of IgG 1, immunoglobulins involved in the allergic processes, not having changes to IgG2a - not involved in these processes. When the spleen cells of OVM sensitized mice with this antigen were incubated with this antigen and later on they measured the amount of OVM-IgE in the supernatant, they noticed that in the mice receiving *Lactobacillus* per os there was a significant reduction of specific IgE without, however, causing changes to the IL levels. These findings suggest that the use of *Lactobacillus*, specifically of the *L. lactis* subsp. Lactis G50 strain, promoted a stimulation of the Th1 line instead of in the Th2, and this is important to consider in the modulation of allergic conditions, since the immune status of humans is closely related to the Th1/ Th2 balance.

Rosenfeldt et al., 2003, reported that there were no significant differences as to the severity of atopic dermatitis in children with overt dermatitis when they used *L. rhamnosus* and *L. reuteri* for 6 weeks when compared to placebo; nonetheless, analyzing the atopic child with a positive prick test and high IgE level there was a reduction in severity, as well as in the levels of cationic eosinophilic protein levels, but without changing the levels of IL-2, IL-4, IL-10 and interferon γ, concluding that there was a positive effect concerning the use of a probiotic^13^.

Peng; Hsu, 2005, carried out a study using strain LP33 *Lactobacillus paracasi*, heat-neutralized, assessing its effects on the improvement of the quality of life of patients with allergic rhinitis induced by house dust. Based on a modified questionnaire of pediatric rhino-conjunctivitis associated with quality of life, and they observed that this means of use was as efficient as the administration of live forms, in quality of life improvements^14^.

Xiao et al., 2006, assessed the use of *Bifidobacterium longum* BB536 during 13 weeks in the evolution of pollinosis from Japanese cedar and, based on the parameters evaluated, they noticed that the use of these probiotic agents did not only improve symptoms, but it also caused a reduction in the TH2 response instead of an increase in TH1 response^15^.

Takahashi et al., 2006, assessed a sequence of DNA nucleotides from the *Bifidobacterium longum*, BB 536 strain probiotic, which inhibited the in vitro production of IgE and it also stimulated the proliferation of B cells, induced the production of IL-12 in a culture of J774.1 and macrophage murine increased the secretion of interferon-Gama, without however having an effect on IL[Bibr bib4][Bibr bib16][Bibr bib17].

Showing a protective effect of probiotics in allergy, Morita et al., 2006, utilized fermented milk with TMC0356 Lactobacillus gasseri in 15 individuals with high serum IgE and allergic rhinitis, in the dose of 200ml/day during 4 weeks and observed a significant decline (p<0.05) in the total IgE serum levels, as well as specific IgE for mite and Japanese cedar pollen, after 28 days of exposure compared to the baseline value; besides these findings, there was an increase in the number of TH1 cells^18^.

The Japanese cedar pollinosis is an IgE-mediated type I allergic reaction, caused by the exposure to *Cryptomeria japonica*, Japanese cedar or Japanese araucaria, which affects 16% of the Japanese population, currently being considered a public health problem in that country^19^.

Ogawa et al., 2006, studied the development of skin lesions similar to that of atopic dermatitis in NC/Nga mice with clinical symptoms and blood parameters which are characteristic of allergic rhinitis in healthy volunteers during the time of pollination in Japan by the *Cryptomeria japonica*, concurrent to the oral administration of a symbiotic combination of *Lactobacillus casei* subsp. *casei* plus dextran, and they noticed that there was an improvement in the skin lesions, although without significant changes as far as the allergic rhinitis goes in these animals. Assessing those patients who used placebo, they observed that there was a trend towards an increase in the levels of specific IgE for this pollen, as well as of cytokines and eosinophils and reduction in the levels of interferon Gama in relation to the individuals who used the probiotic, concluding that, in a certain way, the use of such association helped the individuals have a satisfactory response to rhinitis^20^.

In 2007, Odamaki et al. carried out a clinical trial to assess the effects of BB 536 *Bifidobacterium longum-*enriched in the treatment of Japanese cedar pollinosis and observed that, during the pollination period, the concomitant use of probiotics helped alleviate the symptoms, while the raise in serum levels of eosinophils and a reduction in INFγ were suppressed^19^.

In a new study carried out in 2007, Odamaki et al. observed that, during the pollination period, there was a change in the intestinal microbiota of the individuals, when compared to non-allergic individuals, especially concerning *Bacteroides fragillis*, and the use of a probiotic inhibited this fluctuation, preserving the proper micro flora. The microbiota fluctuation in this study was seen in allergic individuals at the beginning and at the end of the pollination phase, without a concrete scientific explanation, speculating that this could have happened because of the stress caused by these symptoms^21^.

Iwabuchi et al., 2007, studied 17 strains of lactic acid bacteria and 15 of bifidobacteria and observed that in relation to the production of IL12, the former were more efficient. According to the study, the *Bifidobacterium longum* BB536 strain inhibited IL-4 and antigen-specific IgE production, partially independent of the Th1 line cytokines and independent of regulatory cytokines with IL-12, IL10 and TNF-β, which are measured by the antigen-presenting dendritic cells. They also observed that the BB536 stimulated the maturation of dendritic cells, especially after antigen stimulation and which inhibited the TH2 immune response in T memory lymphocytes^22^.

Sunada et al., 2007, assessed the effects of *Lactobacillus acidophilus* L-55 strain in BALB/c mice, with experimental allergic rhinitis and observed that the use of 10mg/day for 4 consecutive weeks resulted in reduced nasal sensitization, the frequency of sneezing after the second week of use, and the control medication reduced these symptoms only on the fourth week. There was also a significant reduction in the specific IgE dosing for OVA (ovalbumin), at the end of 4 weeks, indicating that the administration of L-55 strains of *Lactobacillus* inhibited the IgE production in sensitized mice^23^.

Kawase et al., 2007, used the TMC 0356 strain of *Lactobacillus gasseri* and GG L*actobacillus* in guinea pigs sensitized by the Japanese Cedar pollen, in the dose of 10^3^ CFU during 3 weeks, with the goal of assessing the presence of nasal obstruction induced by the intranasal administration of OVA and changes to the immune cells of the nasal lavage and observed that, in relation to air flow resistance, after nasal administration of Ovalbumin, there was an increase in the resistance in both guinea pigs; however, in those who received *Lactobacillus*, this resistance was lower when compared to the control group after 10 minutes of OVA administration, and these data were statistically significant. In relation to the cells, there was a reduction in the number of leucocytes, especially eosinophils and neutrophils, in the nasal lavage and serum reduction in the levels of specific OVA-IgE levels and, despite this data not being significant in the study, they suggest a considerable action of these *Lactobacillus*, thus requiring further studies for such confirmation^24^.

Rasche et al., 2007, co-stimulated peripheral mononuclear cells of individuals allergic to grass pollen (n=10) and those non-allergic (n=19) with inactive *Lactobacillus acidophilus* and the non-pathogenic Nissle strain of *Escherichia coli* and observed that both bacteria modulated the immune response from changes to the CD23 and expression of co-stimulatory molecules. In relation to the production of cytokines, there was a response depending on the presence or not of atopy; however there is an increase in the Th1 cells of atopic individuals^25^.

A lysate of Enterococcus faecallis FK-23 (LFK), a probiotic product of *E faecallis*, showed an inhibitory effect on the local buildup of eosinophils induced by allergens in active skin anaphylaxis in rat models. By the same token, a pilot clinical study in humans showed that the number of eosinophils in the peripheral blood was significantly reduced after treatment with LFK in patients with permanent allergic rhinitis. Therefore, it has been suggested that LFK supplementation can play a role in the prevention and/or in the Th2 predominance transfer to a Th1/Th2 balance^26^.

Considering the above, Shimata et al., 2007, investigated the effects of using oral antibiotics in weaned mice, and the influence of oral consumption either associated or not to LFK (FK-23 Enterococcus faecallis lysate) on the local buildup of allergy-induced eosinophils, the levels of total IgE, antigen-specific IgE and IgG2a in the serum and on the intestinal micro flora. For the study they used 3-week-old Balb/Cmice sensitized with the allergen (Japanese Cedar pollen) experimental model and treated with macrolide and macrolide + *Enterococcus faecallis* FK-23 (LFK) lysate and observed that the *Lactobacilli* species were distinctly eliminated in the mice exposed to erythromycin on the 7th day and totally recovered in the mice treated with LFK-erythromycin on the 28th day, but not found on the mice which used erythromycin without LFK. They did not observe a statistically significant difference between the mice with and without LFK in terms of peritoneal buildup of eosinophils and specific serum IgE and IgG2a; however, in the total IgE and IgG2a ratio, there was a significant increase in the mice which used erythromycin only when compared to those who used LFK ^26^.

Giovannini et al., 2007, assessed the benefits of a prolonged use, 12 months, of fermented milk, with one species of *Lactobacillus casei* in 187 children between 2 and 5 years with allergic rhinitis and/or asthma, and observed that there was a significant improvement in the number of rhinitis episodes per year; however, without change on the asthma evolution^27^.

Kuitunen et al., 2008, administered a mixture of probiotics (*Lactobacilllus, Bifidobacteria and Propionibacteria)* or placebo in pregnant women in the last gestational months and in their babies, with a high risk of allergy, up to 6 months of life, and these children were evaluated at 5 years of age. The authors did not find statistically significant differences concerning the incidence of allergic diseases such as eczema, rhinitis, asthma and food allergies in the group receiving probiotics who were born out of c-sections^28^.

Dev et al., 2008, evaluated the action of Lac-B, which is a mix of *Bifidobacterium infantis* and *Bifidobacterium longum* dried-frozen, on the release of histamine in a dry experimental model with rats with allergic rhinitis and observed that the use of these acid bacteria promoted a reduction in the expression of H1 receptors on the mucosa as well as RNAm expression of the decarboxylase histidine, reducing the release and action of histamine and alleviating the allergic symptoms. In this study the pretreatment with Lac-B also reduced RNAm expression of IL-4 and IL-5 without; however, being statistically significant when compared to the control individuals. This data reinforce the action of these bacteria on the inhibition of the cytokines responsible for the allergic reaction.^29^

Vliagoftis et al., 2008, carried out a review study using Pubmed database on the use of probiotics in the treatment of allergic rhinitis and asthma, and they observed that its use reduce the severe forms of allergic rhinitis and reduce the use anti-allergic drugs, without studies which show a satisfactory response as far as asthma is concerned^30^.

Ivory K et al., 2008 observed through a doubleblind placebo-controlled study that the five-month use of *Lactobacillus casei Shirota (LcS)* caused a significant reduction in the levels of antigen-induced IL-5 and IL-6 and interferon Gama (IFN-γ) compared to placebo and IgG increase without impact on IgE reduction^31^.

## DISCUSSION AND CONCLUSION

Despite significant studies, very little is known about the use of these substances and the effects of extrinsic factors which would change the intestinal microbiota and promote diseases - allergic rhinitis among them.

Since allergic rhinitis remains a constant challenge for physicians, it is necessary to search for new treatment alternatives which could improve the quality of life of patients, and probiotics could represent a promising alternative. Nonetheless, more encompassing studies, with more patients, need to be carried out, in such a way that probiotics can be fully used, enabling a better treatment approach with benefits for the patients.

All these studies showed that probiotics promote an improvement to the immune response of the human body, and we noticed a satisfactory action on the modulation of the allergic response.

Based on probiotics and allergic rhinitis studies, we may conclude that the use of these substances brought about a satisfactory response in symptom control, better quality of life and lab markers both *in vitro* and *in vivo*.
